# The Gut Microbial Metabolite Indole-3-Acetic Acid Reprograms Systemic Homeostasis and Ameliorates IBD-Associated Cachexia Independent of Food Intake

**DOI:** 10.3390/ijms262311260

**Published:** 2025-11-21

**Authors:** Ayame Tomii, Chihiro Takei, Keisuke Yoshikiyo, Hidehisa Shimizu

**Affiliations:** 1 Graduate School of Natural Science and Technology, Shimane University, 1060 Nishikawatsu-Cho, Matsue 690-8504, Shimane, Japan; 2 Faculty of Life and Environmental Sciences, Shimane University, 1060 Nishikawatsu-Cho, Matsue 690-8504, Shimane, Japan; 3 The United Graduate School of Agricultural Sciences, Tottori University, 4-101 Koyama-Minami, Tottori 680-8553, Tottori, Japan; 4 Institute of Agricultural and Life Sciences, Academic Assembly, Shimane University, 1060 Nishikawatsu-Cho, Matsue 690-8504, Shimane, Japan; 5 Estuary Research Center, Shimane University, 1060 Nishikawatsu-Cho, Matsue 690-8504, Shimane, Japan; 6 Interdisciplinary Center for Science Research, Shimane University, 1060 Nishikawatsu-Cho, Matsue 690-8504, Shimane, Japan

**Keywords:** gut microbiota, microbial metabolite, cachexia, anemia of inflammation, gut–kidney axis, systemic complications, principal component analysis

## Abstract

Inflammatory bowel disease (IBD) is associated with severe systemic complications, including cachexia, anemia, and renal dysfunction, which represent a significant unmet medical need. The gut microbial metabolite indole-3-acetic acid (IAA) is known to be reduced in IBD; however, its therapeutic potential remains unclear. This study aimed to determine whether oral supplementation with IAA could ameliorate intestinal inflammation and its associated systemic complications. Using a dextran sulfate sodium (DSS)-induced colitis mouse model, we administered oral IAA and evaluated a comprehensive panel of clinical, metabolic, renal, and hematological parameters. Systemic health status was assessed using Principal Component Analysis (PCA). IAA administration significantly ameliorated DSS-induced colitis, reducing the Disease Activity Index (DAI) (3.88 vs. 3.13; *p* < 0.05) and significantly attenuating colon shortening (5.0 cm vs. 5.78 cm; *p* < 0.05) compared to the DSS-alone group. Crucially, it markedly suppressed systemic complications: IAA ameliorated DSS-induced cachexia (ΔBody weight, −3.27 g vs. −1.83 g; *p* < 0.05), an effect independent of food intake (N.S.). Furthermore, IAA mitigated early-stage renal dysfunction, as evidenced by a significant reduction in plasma Creatinine (Cr) levels (0.12 mg/dL vs. 0.10 mg/dL; *p* = 0.05), and reversed the decline in plasma iron levels associated with anemia (45.75 μg/dL vs. 63.50 μg/dL; *p* < 0.05). PCA revealed that IAA induced a distinct recovery profile, significantly improving the systemic health index without fully restoring the original homeostatic state. Oral IAA exerts pleiotropic effects on both intestinal inflammation and systemic complications. Its food intake-independent anti-cachectic mechanism represents a novel therapeutic paradigm for IBD-associated wasting. These findings position IAA as a promising candidate for microbial metabolite-based therapy aimed at reprogramming, rather than merely restoring, systemic homeostasis in IBD.

## 1. Introduction

Inflammatory Bowel Disease (IBD) is a chronic inflammatory disorder of the gastrointestinal tract [1]. However, over the past decades, IBD has been increasingly recognized as a systemic disease that affects nearly every organ system [2]. Recent studies have indicated that approximately 30–50% of patients develop diverse extraintestinal manifestations (EIMs), including anemia, malnutrition, liver disorders such as primary sclerosing cholangitis, and renal dysfunction [1,3,4]. These complications significantly impair the quality of life and prognosis of patients, posing major challenges to clinical management [1,4]. Moreover, large-scale systematic reviews have revealed that patients with IBD are at a substantially higher risk of serious cardiovascular events, such as myocardial infarction and stroke [5,6,7], suggesting a close link between IBD pathophysiology and systemic conditions.

This gut–systemic connection is not unique to IBD. For example, the gut microbial metabolite indole-3-acetic acid (IAA) has been shown to suppress liver inflammation in mouse models of non-alcoholic fatty liver disease (NAFLD) [8,9] and modulate chemotherapy efficacy in pancreatic cancer patients [10,11,12], indicating its involvement in a wide range of systemic pathologies.

Although IBD was historically considered a disease of Western countries, its incidence has been rising rapidly in newly industrialized nations across Asia and Latin America, emerging as a global public health concern [13,14]. In particular, the prevalence of IBD in Japan, Hong Kong, and the United States is projected to increase further over the next decade, underscoring the urgent need for novel therapeutic strategies [15].

Recent advances in biologics and small-molecule therapies have significantly improved IBD treatment outcomes [1,16,17,18]. Nevertheless, many patients remain inadequately managed [16], and a considerable proportion fail to achieve a satisfactory clinical response [16,17,19]. This issue is compounded by the lack of a standardized definition for “treatment failure.” This ambiguity leads to inconsistent clinical decision-making, such as the timing of switching biologics, and complicates the interpretation of clinical trial outcomes [20,21]. Importantly, current gut-targeted therapies have not been shown to directly address systemic complications, such as cachexia, anemia, and renal dysfunction [1,3,14], highlighting a critical unmet medical need.

The growing recognition of gut microbiota disruption, a condition known as dysbiosis, as a key driver of IBD pathogenesis offers a promising avenue for therapeutic innovation. Accordingly, microbiota and their metabolites have gained attention as novel therapeutic targets [1,22,23,24]. Established animal models, such as the dextran sulfate sodium (DSS)-induced colitis mouse model, have been instrumental in advancing IBD research [1,22,23,24]. Notably, germ-free (GF) mice exhibit milder colitis in these models, emphasizing the role of the microbiome in disease progression [1,16,22]. Clinical trials have further demonstrated that specific bacteria and metabolites influence therapeutic responses to fecal microbiota transplantation (FMT), a procedure that restores a healthy microbial balance by transferring stool from a healthy donor, suggesting that microbiota manipulation may be a viable strategy [16,22,25].

Dysbiosis is also known to affect tryptophan metabolism, leading to reduced production of IAA, one of its major microbial metabolites [26,27]. A previous metabolomic study using a DSS-induced colitis model reported a significant decrease in colonic IAA levels during the acute phase of inflammation [26,27], implicating IAA deficiency in the exacerbation of IBD pathology.

Based on these findings, we hypothesized that oral supplementation with IAA would exert pleiotropic protective effects not only against local intestinal inflammation but also against systemic complications such as cachexia and renal dysfunction. The present study aimed to determine whether IAA could ameliorate intestinal dysfunction and simultaneously suppress associated systemic complications, including weight loss, adipose tissue depletion, renal dysfunction, and reduced plasma iron levels, in a DSS-induced colitis mouse model.

## 2. Results

### 2.1. IAA Reduces the Severity of Clinical Colitis

**Figure 1:**
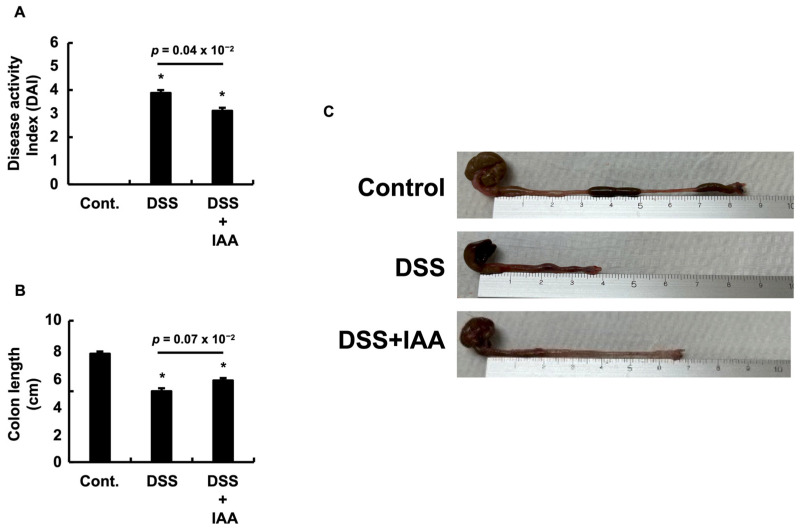
**IAA ameliorates the clinical manifestations of DSS-induced colitis.** (**A**) Disease Activity Index (DAI) scores on day 6 of the experiment. (**B**) Colon length measured at necropsy. (**C**) Representative images showing the macroscopic appearance of the colon in each experimental group. Data are presented as the mean ± SE (*n* = 8 per group). Asterisks denote statistical significance compared to the control group (* *p* < 0.05). The *p*-value for the comparison between the DSS and DSS + IAA groups is also shown. Control (Cont.), dextran sulfate sodium (DSS), DSS with indole-3-acetic acid (DSS + IAA).

To evaluate the therapeutic effects of IAA in a DSS-induced colitis model, we assessed the disease activity index (DAI) and colon length at the time of necropsy. DSS administration resulted in a significant increase in DAI scores compared to the control group (Figure 1A). In contrast, co-administration of IAA (DSS + IAA) significantly reduced the DAI score compared with that in the DSS-alone group. Similarly, DSS treatment caused marked colon shortening relative to the controls, an effect that was significantly attenuated by IAA co-treatment (Figure 1B). Representative macroscopic images of the colon from each group corroborated these quantitative findings (Figure 1C). Collectively, these results indicate that IAA effectively alleviates the clinical manifestations of DSS-induced colitis.

### 2.2. IAA Ameliorates Anorexia and Dehydration During Acute Colitis

**Figure 2:**
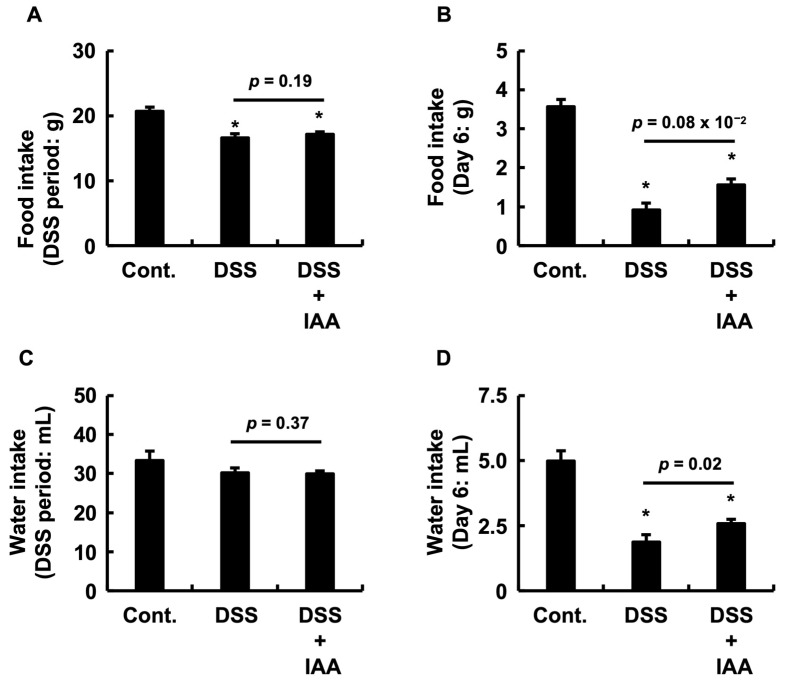
**IAA mitigated the reduction in food and water intake during the acute phase of colitis.** (**A**) Total food intake during DSS administration period. (**B**) Food intake on day 6 of DSS treatment. (**C**) Total water intake during DSS administration period. (**D**) Water intake on day 6 of DSS treatment. Data are presented as the mean ± SE (*n* = 8 per group). Asterisks (*) denote statistically significant differences from the Control group (* *p* < 0.05). The *p*-value represents the comparison between the DSS and DSS + IAA groups. Cont., control; DSS, dextran sulfate sodium; DSS + IAA, DSS with indole-3-acetic acid.

To assess the systemic effects of DSS-induced colitis and to estimate IAA consumption, we measured food and water intake throughout the administration period, with a particular focus on day 6. Throughout the treatment period, no significant differences were observed in total food consumption (Figure 2A) or total water consumption (Figure 2C) between the DSS and DSS + IAA groups. Based on this average water intake (Figure 2C), the average daily IAA consumption in the DSS + IAA group was estimated to be 1249.8 ± 37.1 mg/kg/day. However, on day 6, when inflammation was most pronounced, the DSS group exhibited a significant reduction in both food and water intakes compared to the control group. In contrast, co-administration of IAA significantly ameliorated these reductions compared to the DSS-alone group (Figure 2B,D). These findings indicate that IAA mitigates systemic wasting conditions, such as anorexia and reduced fluid intake, during the acute phase of DSS-induced colitis. Crucially, the significantly maintained water intake on day 6 (Figure 2D) demonstrates that the DSS + IAA group continued to consume IAA effectively, even during the most severe phase of the disease, directly addressing the potential concern over reduced intake due to illness.

### 2.3. IAA Attenuates Weight Loss and Adipose Tissue Depletion in DSS-Induced Colitis

**Figure 3:**
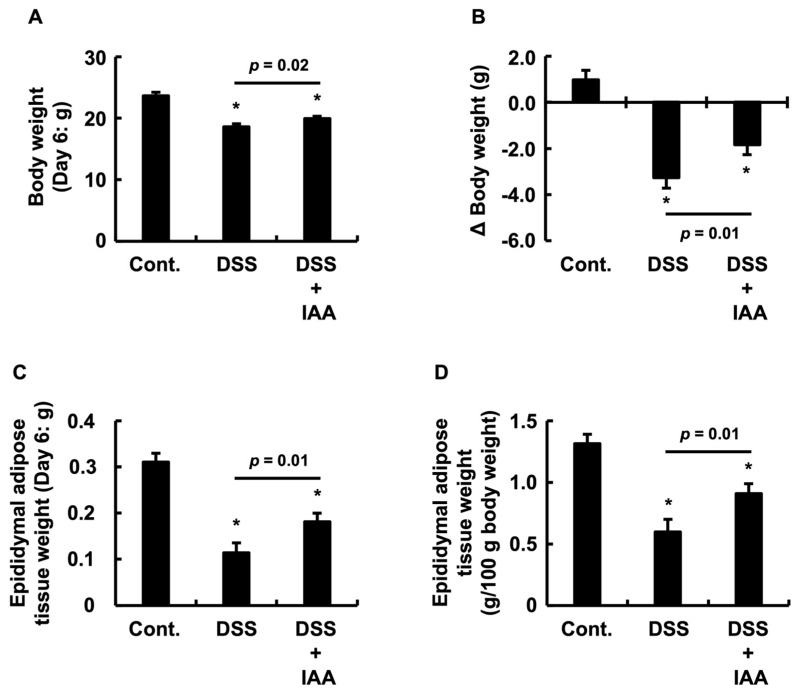
**IAA mitigated systemic wasting associated with DSS-induced colitis.** (**A**) Final body weights on day 6. (**B**) Change in body weight (ΔBody weight) from the start of DSS administration period. (**C**) Absolute mass of epididymal adipose tissue on day 6. (**D**) Relative mass of epididymal adipose tissue per 100 g body weight. Data are presented as the mean ± SE (*n* = 8 per group). Asterisks (*) denote statistically significant differences from the Control group (* *p* < 0.05). The *p*-value represents the comparison between the DSS and DSS + IAA groups. Cont., control; DSS, dextran sulfate sodium; DSS + IAA, DSS with indole-3-acetic acid.

To evaluate the effect of IAA on systemic catabolism (wasting) in DSS-induced colitis, we measured changes in body weight and epididymal adipose tissue mass. While the control group gained weight over the experimental period, the DSS group exhibited marked body weight loss. However, IAA co-administration significantly attenuated DSS-induced weight loss (Figure 3A). A similar result was observed for the change in body weight (ΔBody weight) (Figure 3B). Furthermore, compared to the control group, the DSS group showed a significant reduction in both the absolute and relative (as a ratio to body weight) mass of the epididymal adipose tissue. In the DSS + IAA group, this loss of adipose tissue was significantly ameliorated in terms of both absolute and relative values (Figure 3C,D). These results indicate that IAA mitigates systemic wasting associated with DSS-induced colitis.

### 2.4. IAA Mitigates Incipient Renal Dysfunction in DSS-Induced Colitis

**Figure 4:**
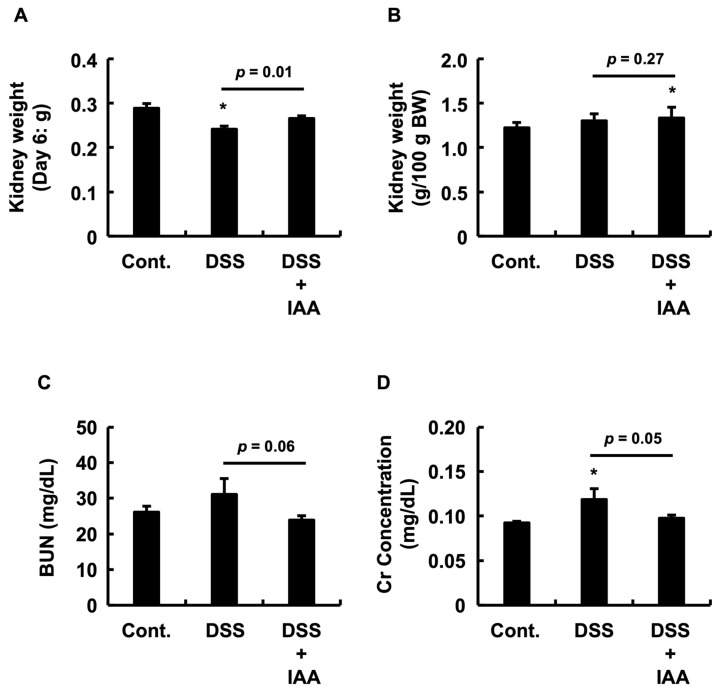
**IAA attenuates incipient renal dysfunction in DSS-induced colitis.** (**A**) Absolute kidney weights at necropsy. (**B**) Relative kidney weight per 100 g body weight. (**C**) Plasma creatinine (Cr) concentration. (**D**) Plasma blood urea nitrogen (BUN) concentration. Data are presented as the mean ± SE (*n* = 8 per group). Asterisks (*) indicate statistically significant differences compared to the Control group (* *p* < 0.05). The *p*-value for the comparison between the DSS and DSS + IAA groups is also shown. Cont., control; DSS, dextran sulfate sodium; DSS + IAA, DSS with indole-3-acetic acid.

To assess the renal impact of DSS-induced colitis, we measured the kidney mass and plasma markers of renal function. DSS treatment caused a significant reduction in absolute kidney mass compared to that in the control group, an effect that was significantly attenuated by the co-administration of IAA (Figure 4A). Regarding relative kidney mass (normalized to body weight), no significant difference was observed between the DSS and control groups; however, the DSS + IAA group showed a significantly higher relative mass than the control group (Figure 4B). Furthermore, DSS treatment tended to elevate plasma creatinine (Cr) and blood urea nitrogen (BUN) levels (Figure 4C,D). IAA co-treatment significantly lowered plasma Cr compared to the DSS group, while the reduction in BUN was not statistically significant.

### 2.5. IAA Specifically Reverses Plasma Iron Depletion in DSS-Induced Colitis

**Figure 5:**
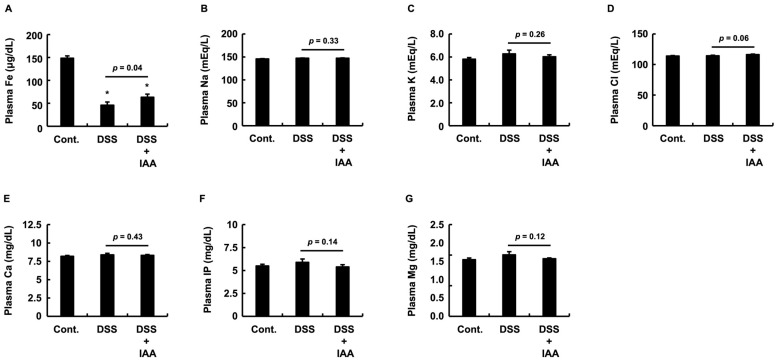
**IAA specifically ameliorated the reduction in plasma iron concentration.** Plasma concentrations of (**A**) iron (Fe), (**B**) sodium (Na), (**C**) potassium (K), (**D**) chloride (Cl), (**E**) calcium (Ca), (**F**) inorganic phosphorus (IP), and (**G**) magnesium (Mg). Data are presented as the mean ± SE (*n* = 8 per group). Asterisks (*) indicate statistically significant differences compared to the Control group (* *p* < 0.05). The *p*-value for the comparison between the DSS and DSS + IAA groups is also shown. Cont., control; DSS, dextran sulfate sodium; DSS + IAA, DSS with indole-3-acetic acid.

To investigate systemic biochemical alterations in DSS-induced colitis, we measured plasma concentrations of various minerals and electrolytes. The most prominent change was observed in the plasma iron (Fe) levels. DSS treatment caused a significant decrease in plasma iron levels compared to that in the control group, and this reduction was significantly ameliorated by the co-administration of IAA (Figure 5A). In contrast, no significant differences were observed in the plasma concentrations of sodium (Na), potassium (K), calcium (Ca), inorganic phosphorus (IP), or magnesium (Mg) between the DSS and DSS + IAA groups (Figure 5B,C,E–G). Plasma chloride (Cl) levels tended to increase with DSS treatment, but the suppressive effect of IAA was not statistically significant (Figure 5D). Taken together, these findings indicate that IAA specifically ameliorates the dysregulation of plasma iron metabolism in DSS-induced colitis.

### 2.6. Integrative Assessment of Systemic Pathological State

**Figure 6:**
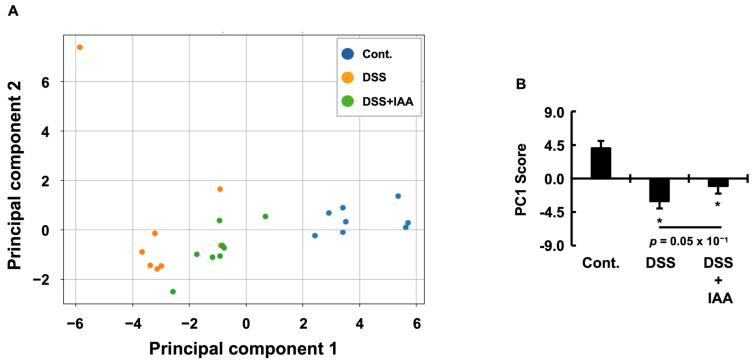
**Integrative assessment of the systemic pathological state using Principal Component Analysis (PCA).** (**A**) PCA score plot showing the distribution of individual mice from the Cont., DSS, and DSS + IAA groups in the space defined by the first two principal components (PC1 and PC2). Each point represents one mouse. (**B**) Bar graph showing the mean scores for principal component 1 (PC1) in each group. PC1 serves as an integrated index of systemic health, with higher scores indicating a healthier state of health. Data are presented as the mean ± SE (*n* = 8 per group). Asterisks (*) indicate statistically significant differences compared to the Control group (* *p* < 0.05). The *p*-value for the comparison between the DSS and DSS + IAA groups is also shown. Cont., control; DSS, dextran sulfate sodium; DSS + IAA, DSS with indole-3-acetic acid.

To conduct a multifaceted evaluation of the therapeutic effects of IAA, we applied Principal Component Analysis (PCA) to the dataset from our DSS-induced colitis mouse model. The analysis effectively captured the principal biological variations within the complex dataset, with the first two principal components (PC1 and PC2) explaining 65.1% of the total variance (Table 1). The first principal component (PC1), which accounted for 49.4% of the total variance, was characterized by a strong negative loading from the Disease Activity Index (DAI) and strong positive loadings from colon length and body weight gain. Based on these loadings, we defined PC1 as a “disease severity axis” that integrates IBD pathology with the overall systemic health status (Table 2). On this axis, a higher score indicates a healthier state, whereas a lower score indicates a more severe disease. The second principal component (PC2), which explained 15.8% of the variance (Table 1), was primarily driven by markers related to kidney function and metabolic status, such as blood urea nitrogen (BUN) and plasma magnesium concentrations. Accordingly, we interpreted PC2 as a “metabolic and renal function axis,” representing sarcopenia-related complications independent of local intestinal inflammation (Table 3). To statistically validate the group separation suggested by the PCA score plot (Figure 6A), we performed multivariate analysis of variance (MANOVA). The results revealed a highly significant difference among the experimental groups when considering PC1 and PC2 (Table 4). Subsequent multivariate pairwise comparisons using Hotelling’s T^2^ test confirmed that all group pairs were significantly different in the principal component space (Table 5). Notably, the overall phenotype of the IAA-treated group significantly improved compared to that of the DSS group. We then focused on the primary axis of variation, PC1, and conducted a one-way analysis of variance (ANOVA), which similarly confirmed a strong significant difference between the groups (Table 6). The PC1 score of the DSS group was significantly lower than that of the control group, and this score significantly improved following IAA administration. However, the PC1 score of the IAA group remained significantly lower than that of the control group, indicating that IAA exerts a partial, yet meaningful therapeutic effect (Figure 6B). In contrast, no significant differences were found in the mean PC2 (“metabolic and renal function axis”) scores among the groups by ANOVA (Table 6). Nevertheless, while the DSS group included outlier individuals with extremely high PC2 scores, no such outliers were observed in the IAA group, suggesting that IAA may help prevent the development of severe systemic complications (Figure 6A).

### 2.7. IAA Remodels the Physiological Correlation Network Disrupted by DSS-Induced Colitis

To understand the systemic effects of IBD and the therapeutic mechanisms of IAA at the system level, we analyzed the changes in the correlation network structure among the physiological parameters for each group. A network was constructed using Spearman correlation analysis, and significant changes in correlations between groups (Control vs. DSS, Control vs. IAA, and DSS vs. IAA) were statistically validated using Fisher’s Z-transformation and Z-difference test (Table 7). The results revealed that each group formed qualitatively distinct correlation networks. In the control group, a harmonious “homeostasis network” was observed, in which body weight and nutrient intake served as central hubs that were strongly and positively correlated with a cluster of variables representing the metabolism and mineral balance. In contrast, this network collapsed in the DSS group and was replaced by a pathological “disease network,” in which the Disease Activity Index (DAI) became the central hub, exhibiting strong negative correlations with multiple health indicators, such as body weight, colon length, and fat mass. In the IAA group, the influence of the DAI hub was markedly weakened, resulting in a transitional “recovery network” with characteristics distinct from both healthy and diseased states. We interpreted these shifts in correlation strength as indicators of disease-induced disruption or treatment-induced remodeling of the physiological relationships. Quantitative evaluation using the Z-difference test confirmed that this “rewiring” of the system’s correlation architecture was significant. For example, the strong positive correlation between body weight and inorganic phosphorus (IP) observed in the control group was completely lost in the DSS group, representing a significant collapse of the metabolic linkage. Furthermore, therapeutic rewiring by IAA was clearly demonstrated; the aberrant positive correlation between final water intake and colon length observed in the DSS group was entirely abolished in the IAA group, and this dissolution of the pathological link was also statistically significant. Interestingly, recovery induced by IAA did not necessarily represent reversion to the original healthy state. For instance, the relationship between weight gain and calcium (Ca), which was strongly positive in the control group, was significantly inverted to a negative correlation in the IAA group. This suggests that the post-treatment recovery phase operates under distinct physiological regulation—“new normal”—that differs from the original homeostatic state.

## 3. Discussion

In the present study, we investigated the pleiotropic therapeutic effects of the gut microbial metabolite IAA in a mouse model of IBD. Our findings demonstrate that the efficacy of IAA extends beyond the suppression of intestinal inflammation to ameliorating systemic complications, including cachexia, inflammatory anemia, and incipient renal dysfunction. Notably, we discovered that IAA ameliorates cachexia through a food intake-independent mechanism. This therapeutic effect was observed despite the lack of complete recovery in total food consumption, indicating that IAA directly targets the host’s underlying metabolic dysregulation rather than merely improving nutritional status. This discovery is particularly significant as it offers a novel therapeutic approach for wasting conditions in patients, which are often refractory to conventional nutritional support. By addressing the core metabolic pathology, IAA represents a potential paradigm shift in the management of IBD-associated cachexia and highlights a promising strategy for addressing the critical unmet need for treatments targeting systemic complications of IBD.

The pleiotropic effects of IAA on both intestinal inflammation and systemic complications in IBD, as demonstrated in this study, were integratively captured by PCA. This analysis consolidated all measured physiological variables, and the resulting score plot revealed a distinct shift in the IAA-treated group away from the diseased state (DSS group) and toward a healthy state (control group). This indicates that IAA exerts a broad restorative influence not only on individual parameters in isolation but also on the physiological network as a whole. Of particular note is the potency of this therapeutic effect. The PC1 score, an integrated index of systemic health, was significantly improved in the IAA-treated group compared to that in the DSS group. However, this recovery did not fully reach the level of healthy controls, as a statistically significant difference remained. This provides compelling evidence that IAA induces robust, albeit partial, normalization of the pathological state. Importantly, this recovery does not represent a simple reversion to the original homeostatic conditions. Although PC1 scores improved, multivariate analysis using Hotelling’s T^2^ test, which incorporated both PC1 and the renal-metabolic axis (PC2), confirmed that the overall phenotype of the IAA group remained statistically distinct from that of the healthy controls. This finding suggests that IAA guides the system toward a unique recovery profile characterized by the activation of distinct compensatory and reparative pathways rather than merely restoring the original state. A deeper analysis of the variable loadings for PC1 provides insights into this unique profile. In the control group, systemic health was primarily defined by fundamental physiological functions, such as food intake and kidney mass. In contrast, the health status of the DSS group was dominated by pathological indicators, namely DAI and loss of adipose tissue. Strikingly, the profile of the IAA-treated group was dramatically altered; while the negative influence of DAI remained, preserved adipose tissue and normalized plasma iron levels emerged as key health indicators. These findings suggest that IAA’s therapeutic efficacy extends beyond anti-inflammatory action to encompass the powerful activation of ‘active reparative processes,’ including the rebuilding of adipose tissue and restoration of iron metabolism. The fact that adipose tissue loss was a key driver of pathology in the DSS group, whereas its preservation became a key factor in the recovery of the IAA group, underscores IAA’s direct role in ameliorating adipose tissue dysfunction—one of the most critical discoveries of this study. This distinct recovery profile suggests that IBD pathophysiology is not monolithic but rather a complex interplay of inflammatory and metabolic dysfunction. Accordingly, our findings support the potential for stratifying patients with IBD into subtypes, such as “inflammation-dominant” versus “metabolic-wasting-dominant” phenotypes, a concept that could inform future personalized therapeutic strategies for patients with IBD. Finally, the emergence of this “reprogrammed” physiological state raises a pivotal question for translational research: Does this represent a stable and resilient form of recovery, or is it a compensatory state that carries potential long-term risks, such as altered metabolic regulation? Elucidating the long-term trajectory of IAA-induced homeostasis is a pivotal next step for its clinical translation.

The mechanisms underlying the amelioration of intestinal inflammation by indole-3-acetic acid (IAA) are multifaceted and extend beyond the canonical aryl hydrocarbon receptor (AhR) pathway. Although IAA is traditionally considered an AhR ligand [10,12,26,27], this pathway alone cannot fully account for its potent therapeutic efficacy in IBD. Notably, AhR expression is markedly downregulated in inflamed tissues of patients with IBD [28,29] and DSS-induced colitis mouse models [30,31], and the binding affinity of IAA for AhR is relatively low compared to that of other endogenous ligands [32]. These dual limitations, receptor downregulation and low ligand affinity, strongly suggest that non-AhR pathways play a predominant role in mediating IAA’s effects. Our previous studies have provided critical insights into these alternative mechanisms. We previously demonstrated that IAA suppresses TNF-α expression in colonic epithelial cells (Caco-2) via an AhR-independent pathway [33]. Furthermore, we identified Toll-like receptor 4 (TLR4) as a novel receptor for IAA [34,35] and showed that its activation leads to downstream stimulation of the JNK signaling cascade [35]. These findings support the hypothesis that IAA’s anti-inflammatory effects in vivo arise from a combination of signaling pathways. In addition to the conventional AhR-dependent mechanism, TLR4-mediated signaling may play a complementary or even dominant role in the pathogenesis of IBD. Additional evidence supports the involvement of diverse AhR-independent mechanisms. We have previously shown that IAA activates ERK in Caco-2 cells [35], and recent studies have confirmed ERK activation in the murine gut, promoting IL-10 production and contributing to mucosal healing [36]. Moreover, IAA activates the Nrf2/HO-1 pathway in macrophages, conferring strong antioxidant and cytoprotective effects [37]. Collectively, these findings suggest that the pleiotropic therapeutic efficacy of IAA is mediated by a network of signaling pathways, including AhR, TLR4-JNK, ERK, and Nrf2/HO-1, rather than a single canonical route. This mechanistic diversity may underlie IAA’s robust anti-inflammatory action and its ability to modulate complex disease phenotypes such as IBD.

A pivotal finding of this study is that IAA ameliorates cachexia through a dual mechanism of action. First, despite the absence of a significant recovery in total food intake over the experimental period, IAA markedly suppressed both body weight loss and reduction in adipose tissue mass. This strongly suggests that IAA exerts its primary therapeutic effect on systemic wasting via a mechanism independent of food intake. This distinction is critical. The wasting observed in the DSS group cannot be attributed solely to anorexia (reduced food intake). The accompanying disproportionate loss of adipose tissue (Figure 3C,D)—a loss far exceeding what would be expected from caloric restriction alone—is a definitive hallmark of inflammatory cachexia (i.e., hypercatabolism). IBD-associated cachexia is driven not only by anorexia but also by metabolic dysregulation, particularly hypercatabolism induced by pro-inflammatory cytokines. Our data indicate that IAA may directly counteract this dysregulated metabolic state, even under conditions of insufficient nutritional intake, offering a novel therapeutic approach that targets the core pathophysiology of cachexia. The regulation of energy metabolism within adipose tissue likely plays a central role in this metabolic modulation [38,39,40]. Supporting this, PCA loading analysis revealed that adipose tissue mass was a major positive contributor to systemic health (PC1 score), highlighting its importance in disease recovery. Therefore, we propose that IAA suppresses systemic catabolism by directly preventing adipose tissue dysfunction. Second, during the late stage of the disease, when pathology was most severe, IAA partially alleviated anorexia, leading to a significant recovery in food and water intake. This secondary effect is critical for breaking the vicious cycle of acute exacerbations: inflammation → anorexia → nutritional decline → immune impairment → further inflammation. The restoration of feeding behavior likely provided nutritional support for recovery, mediated by an overall improvement in systemic conditions. Taken together, IAA combines direct pharmacological action with a subsequent indirect effect mediated by systemic improvement, forming a multifaceted therapeutic profile. This dual action can be understood within the framework of the “gut–adipose axis vicious cycle,” a concept that is gaining increasing recognition. In this model, gut dysbiosis and intestinal inflammation initiate adipose tissue dysfunction, where fat acts not only as an energy depot but also as an endocrine organ secreting adipokines (gut → fat axis) [38,39,40,41]. This link is supported by studies showing that microbiota from DSS-treated mice disrupt fat metabolism when transplanted into germ-free mice [42]. Dysfunctional adipose tissue releases pro-inflammatory cytokines that exacerbate intestinal inflammation (fat → gut axis), creating a self-amplifying loop. We propose that IAA acts as a “circuit breaker” for this pathological loop by simultaneously ameliorating both intestinal inflammation and systemic cachexia. Specifically, IAA may suppress adipose tissue inflammation by modulating macrophage polarization from pro-inflammatory M1 phenotype to an anti-inflammatory M2 phenotype. Additionally, IAA may normalize adipocyte function by restoring the secretion of beneficial adipokines, such as adiponectin, while suppressing catabolic mediators.

Our finding that IAA markedly ameliorates inflammatory anemia provides new insights into the clinical dilemma known as the “iron paradox” in IBD treatment. Anemia is among the most common and debilitating complications of IBD; however, its management remains a major clinical challenge [43,44]. Although oral iron supplementation is widely used, it carries the risk of exacerbating intestinal inflammation due to the induction of oxidative stress in the gut [45,46,47,48]. Indeed, high-dose oral iron has been shown to aggravate colonic damage in DSS-induced colitis models [47]. In contrast, systemic iron repletion via routes such as intraperitoneal injection can restore iron levels without direct gut exposure and has been reported to enhance intestinal barrier integrity [49]. IAA offers a promising solution to this therapeutic paradox. The regulation of hepcidin, the master hormone of iron metabolism, is complex in the context of DSS-induced colitis and cannot be explained by a simple inflammation-driven increase. Key inflammatory cytokines such as TNF-α [50], as well as erythroferrone, a hormone secreted in response to bleeding, can suppress hepatic hepcidin production as a compensatory mechanism [51]. The severe anemia observed in the DSS group likely reflects massive iron loss due to intestinal bleeding, which overwhelms this compensatory suppression. Importantly, the therapeutic effect of IAA on anemia appears to extend beyond its systemic anti-inflammatory action. Hepcidin not only regulates systemic iron homeostasis but also plays a local role in intestinal tissue repair, with its production by conventional dendritic cells (cDCs) in the gut being potently induced by microbial stimuli [52]. As a direct microbial metabolite, IAA may act as a stimulus, suggesting that it functions not only as an anti-inflammatory agent but also as a pro-reparative signal that activates intrinsic mucosal healing pathways via dendritic cells. We propose that the anti-anemic effect of IAA is rooted in a dual mechanism: locally, it suppresses intestinal bleeding by promoting tissue repair, and systemically, it improves iron utilization by reducing inflammation. Supporting this, our study found that IAA selectively restored plasma iron concentrations, whereas other minerals, including sodium, potassium, calcium, phosphorus, magnesium, and chloride, remained unaffected. This specificity suggests that IAA targets iron metabolism without disrupting the overall mineral balance, which is a clinically advantageous feature for therapeutic applications.

The data from this study clearly demonstrate that adipose tissue health and iron metabolism are not independent of each other. Our correlation analysis revealed a significant positive association between adipose tissue mass and plasma iron concentration, providing direct evidence that the suppression of cachexia and amelioration of anemia are intimately linked. At the core of this connection lies systemic inflammation, a shared etiological driver of both pathologies. We propose that adipose tissue, when maintained in a healthy state by IAA, functions as an endocrine organ that suppresses the production of pro-inflammatory cytokines, thereby attenuating the systemic inflammatory milieu. This anti-inflammatory environment likely contributes to the normalization of hepcidin regulation, which is often disrupted in IBD and leads to impaired iron utilization in IBD patients. Through this mechanism, IAA may initiate a positive feedback loop: by preserving adipose tissue integrity, it reduces inflammation, which in turn restores iron metabolism and further supports systemic recovery. The pleiotropic effects of IAA observed in this study may have broader implications beyond IBD. Emerging research suggests that “Gut-derived systemic inflammation,” resulting from intestinal barrier disruption, is a common pathogenic axis in various chronic diseases, including non-alcoholic fatty liver disease (NAFLD), chronic kidney disease (CKD), and cardiovascular disorders. Our findings position IAA as a key molecule capable of interrupting the pathological gut–systemic axis. This insight holds the potential to establish a new therapeutic paradigm not only for IBD but also for other conditions associated with gut dysbiosis, such as metabolic syndrome and chronic low-grade inflammation, which is characteristic of aging (inflammaging). In conclusion, our results strongly suggest that IAA acts as a higher-order regulator of physiological processes. Rather than merely suppressing local intestinal inflammation, it disrupts systemic pathological signaling originating from the compromised gut barrier, thereby restoring homeostasis across the interconnected “gut–adipose–iron metabolism” network of the host. This concept provides a theoretical foundation for a novel therapeutic strategy that views IBD as a disorder of disrupted inter-organ communication and seeks to resolve systemic pathology by targeting the gut environment.

The present study demonstrated that IAA exerts a protective effect against incipient renal dysfunction associated with IBD. This finding highlights the therapeutic potential of targeting the “gut–kidney axis,” a critical component of extraintestinal manifestations of IBD. Recent meta-analyses have shown that IBD significantly increases both the prevalence and incidence of CKD [53,54,55], and a Mendelian randomization study has suggested a genetic causal link between IBD and nephrolithiasis [56], underscoring the pathophysiological relevance of this axis. In this clinical context, the improvement in early-stage renal dysfunction by IAA observed in our study is particularly noteworthy. While IAA is known to accumulate as a protein-bound uremic toxin (PBUT) in patients with advanced CKD, contributing to cardiovascular toxicity and disease progression [57,58], our results suggest a contrasting beneficial role in early-stage pathology. This dichotomy exemplifies the principle of hormesis, wherein a compound exerts protective effects at low or physiological concentrations but becomes toxic at higher concentrations. In the setting of IBD-associated renal dysfunction, the concentrations of IAA achieved through oral supplementation likely fall within a “therapeutic window,” activating protective pathways such as Nrf2 and AhR and exerting anti-inflammatory and antioxidant effects [24,59,60]. In contrast, pathologically elevated levels in advanced CKD may exceed this range, leading to systemic toxicity. This hormetic perspective underscores the importance of defining not only a safe dose but also the optimal therapeutic dose for the clinical application of IAA. The mechanism underlying the renoprotective effects of IAA is likely to be multifactorial. First, the systemic anti-inflammatory effects demonstrated in our study may indirectly reduce the renal inflammatory burden. Second, IAA may act directly on renal tissues, as supported by several molecular pathways. Gut dysbiosis has been implicated in CKD progression, and gut-derived metabolites, such as IAA, may modulate this process [57]. In a calcium oxalate nephrolithiasis model, IAA ameliorated oxidative stress and inflammation via AhR activation and NF-κB suppression [61]. In drug-induced nephrotoxicity, IAA protects the renal tissue by activating the Nrf2/HO-1 pathway [60]. Additionally, our previous work implicated TLR4 signaling in IAA’s anti-inflammatory effects [34,35], which may also contribute to renal protection. Notably, the dissociation between morphological and functional markers observed in our study is of particular importance. While DSS treatment significantly reduced absolute kidney mass, plasma creatinine, and BUN levels showed only modest changes, suggesting that structural alterations precede overt functional decline. This observation provides important clinical insights: in the early stages of renal involvement in IBD, morphological assessments such as renal ultrasound may be more sensitive than conventional biochemical markers. The ability of IAA to prevent kidney atrophy suggests its potential as a prophylactic agent against early-stage renal pathologies.

It has been suggested that the mechanism of action of IAA may not be limited to its direct effects on the host physiology. As part of its role in strengthening the intestinal barrier, IAA has been reported to promote mucin sulfation via AhR activation, thereby enhancing the quality and integrity of the mucus layer [27]. Moreover, a “metabolite cascade” has been described in which IAA serves as a substrate for other gut bacteria, facilitating the production of secondary bioactive compounds, such as R-equol, which exerts additional anti-inflammatory effects [62]. These findings indicate that IAA does not operate through a single pathway but rather acts as a central regulator of gut homeostasis, engaging both host signaling networks and metabolic interactions within the gut microbiota. To fully elucidate the mechanism of action of IAA, a critical question remains regarding its pharmacokinetics: does orally administered IAA act locally within the intestinal lumen, or is it absorbed into the circulation to exert systemic effects on distant organs. For example, the observed amelioration of anemia could result from the local suppression of intestinal bleeding (a mucosal effect), direct regulation of hepatic hepcidin production (a systemic effect), or a combination of both. Addressing this question will require tracer studies using isotopically labeled IAA to determine its absorption, distribution, and site-specific activity. If systemic absorption is limited, the therapeutic effects of IAA may be mediated by indirect signaling mechanisms, such as neural pathways or humoral factors, that transmit signals from the gut to peripheral organs. This implies that IAA plays a role in a complex inter-organ communication network involving the gut, brain, liver, adipose tissue, and kidneys. Although this study revealed the pleiotropic protective effects of IAA, several limitations should be acknowledged. First, it is also important to note that our study was specifically designed to evaluate the prophylactic efficacy of IAA, mimicking a state of metabolite-replete health prior to insult. A limitation of the current study is therefore that our results do not address whether IAA can also act therapeutically (i.e., after the onset of inflammation). Investigating the curative potential of IAA, including the optimal timing and dosage for treating established colitis, represents a critical and important avenue for future research. Second, the study was conducted exclusively in male mice; given the known sex differences in immune and metabolic responses, the effects of IAA in females warrant further investigation. Third, validation in additional colitis models with distinct immunological mechanisms, such as T-cell transfer colitis, would strengthen the generalizability of our findings. Fourth, while our study provides robust data on clinical manifestations (e.g., DAI, colon length) and systemic complications, we did not perform a detailed histopathological or immunohistochemical analysis of the colonic tissue. As our primary focus was the novel, systemic effects of IAA (e.g., cachexia, anemia), a deep histological investigation was considered beyond the scope of this initial study. Such analysis remains an important future task to fully elucidate the local tissue-level mechanisms of IAA’s protection. Finally, future studies should explore the dose-dependent effects of IAA to determine its optimal therapeutic window. Although previous reports have shown that anemia in DSS-induced colitis models closely mirrors the pathophysiology of human IBD [63,64,65], the ultimate clinical utility of IAA must be confirmed in well-designed clinical trials. Such trials are essential to establish the safety, efficacy, and applicability of this approach across diverse patient populations.

The multifaceted effects of IAA demonstrated in this study pave the way for novel therapeutic strategies for patients with IBD. IAA has the potential to simultaneously address several unmet clinical needs: cachexia, for which current treatments remain largely ineffective; inflammatory anemia, which presents a therapeutic dilemma due to the “iron paradox”; and renal complications, the risk of which has been increasingly recognized in recent years. As a naturally occurring microbial metabolite that can be administered orally, IAA offers a promising low-burden option for prophylactic and adjunctive therapies. Previous studies have shown that endogenous IAA levels are reduced in patients with IBD [24,26], providing a strong theoretical rationale for supplementation therapy. Among its effects, the amelioration of inflammatory anemia is particularly noteworthy. Several lines of evidence support the translational relevance of these findings. First, the DSS-induced colitis model used in this study is widely accepted for its reproducibility and ability to replicate iron-restricted erythropoiesis, a hallmark of anemia in human IBD [63,64,65]. Second, hepcidin-mediated disruption of iron metabolism is a conserved biological mechanism across species [22,50]. Third, unlike synthetic compounds, IAA is an endogenous product of the human gut microbiota and is therefore expected to be highly biocompatible [24,26,27]. However, these promising results must be interpreted with caution. Notably, the DSS model does not fully recapitulate the genetic heterogeneity, immunological complexity, or chronic progression of human IBD [22,31,65]. Species-specific differences in drug metabolism and optimal dosing must also be considered. Our study focused on the prophylactic effects of IAA; future studies should assess its therapeutic efficacy in established disease states. Another important consideration is the chemical form of the IAA. While our study used potassium salt, other studies have employed sodium salt [36,66,67]. This distinction appears to be critical for therapeutic efficacy. A recent, comprehensive study [36] using IAA sodium salt at a concentration (25 mM, approx. 4.38 mg/mL) nearly identical to ours (5 mg/mL, approx. 28.5 mM) reported only partial, molecular-level protection in the DSS model. While it improved histological scores and barrier protein expression, it crucially failed to ameliorate key clinical symptoms, including body weight loss, colon shortening, and the Disease Activity Index (DAI) [36]. In stark contrast, our findings demonstrate that IAA potassium salt at this same concentration provides comprehensive therapeutic benefits. Our potassium salt formulation not only replicated the molecular improvements but also dramatically rescued the severe clinical symptoms (DAI, weight loss) and, critically, ameliorated the severe systemic complications of cachexia, anemia, and renal dysfunction. This strongly suggests that the counter-ion (K^+^ vs. Na^+^) is a decisive, and previously overlooked, factor in determining IAA’s bioavailability and efficacy. Sodium salts generally exhibit higher solubility, whereas potassium salts may provide a more gradual and sustained release. We hypothesize that the potassium salt of IAA maintains effective plasma concentrations for longer durations, thereby translating the molecular-level effects (seen with sodium salt) into robust clinical and systemic recovery (seen in our study). This hypothesis, however, requires future validation through direct pharmacokinetic profiling. Given the concerns about IAA accumulation as a uremic toxin in advanced CKD, careful dose optimization and patient stratification are essential. Among microbiota-targeted therapies, IAA supplementation offers unique advantages over probiotics and fecal microbiota transplantation (FMT). Live bacterial therapies face challenges, such as variable engraftment, strain interactions, and host-specific responses. In contrast, direct supplementation with a defined metabolite, such as IAA, provides a clearer mechanism of action and greater control over dosing and quality, representing a major advantage for drug development. Our findings suggest a more refined paradigm for microbiome-based therapy, focusing on replenishing functional outputs rather than manipulating the microbial ecosystem itself. Translating these results into clinical applications will require overcoming several challenges. First, Phase I clinical trials are needed to determine the optimal dosage and safety profile. A critical component of this will be patient stratification, identifying subgroups, such as the “inflammation-dominant” versus “metabolic-wasting-dominant” phenotypes suggested by our PCA, that may benefit most from IAA therapy. To monitor therapeutic efficacy, biomarkers such as ferritin and hepcidin for iron metabolism and dual-energy X-ray absorptiometry (DEXA) scans for body composition may be useful. In the future, IAA should be explored not only as a monotherapy but also as an adjuvant to enhance the efficacy or mitigate the side effects of existing biological agents. The most pressing future objective is the detailed elucidation of IAA’s mechanism of action. Specific research directions include the following:

- Mechanism of Cachexia Amelioration: Investigate the “food intake-independent” effect by analyzing adipokine expression (e.g., ghrelin, leptin, adiponectin) and lipid metabolism genes in adipose tissue. Determine whether IAA acts directly on appetite centers via the brain–gut axis or indirectly via the resolution of inflammation.
- Scope of Renoprotective Effects: Conduct histopathological evaluations of renal tissue, including fibrosis assessment (e.g., Masson’s trichrome staining) and immunohistochemical analysis of injury markers.
- Effects on the Gut Microbiota: Comprehensive microbiome analyses, including 16S rRNA sequencing and metagenomics, will be performed to assess the influence of IAA on microbial composition and function.

By building upon this foundational research and applying machine learning to the multivariate data obtained in our study, the therapeutic strategy for IAA can be advanced toward a personalized medicine approach. Integrative analysis of the gut microbiota, metabolite profiles, and clinical markers may enable patient stratification, prediction of treatment responsiveness, and identification of early warning indicators and optimal intervention timing. The present study provides a roadmap for translating IAA into a clinically viable therapeutic candidate guided by an evidence-based precision medicine approach.

## 4. Materials and Methods

### 4.1. Materials

The following materials were obtained from the indicated suppliers: 3-indoleacetic acid potassium salt (Tokyo Chemical Industry Co., Ltd., Tokyo, Japan); dextran sulfate sodium (molecular weight 36,000–50,000; MP Biomedicals LLC, Burlingame, CA, USA); MF diet (containing 23.2% crude protein, 4.9% crude fat, 5.9% crude ash, 3.3% crude fiber, 54.7% soluble nitrogen-free extract, and 8.1% moisture; Oriental Yeast Co., Ltd., Tokyo, Japan); isoflurane (Pfizer Japan Inc., Tokyo, Japan); heparin (Nacalai Tesque, Inc., Kyoto, Japan); and aprotinin (FUJIFILM Wako Pure Chemical Corporation, Osaka, Japan).

### 4.2. Animal Experiments

All animal experiments and procedures in this study were approved by the Animal Research Committee of Shimane University (approval no. MA4-08-01; Approval Date: 27 February 2023). The experiments were conducted in accordance with the institutional regulations of Shimane University and complied with the Act on Welfare and Management of Animals (Act No. 105) and the relevant national guidelines in Japan. Five-week-old wild-type C57BL/6J male mice were obtained from Japan SLC (Hamamatsu, Japan). Mice were individually housed to minimize individual variations in food and water intake. To reduce stress, transparent cages were placed in close proximity to each other to allow visual contact between the animals. Each cage was equipped with paper bedding and environmental enrichment to encourage nesting behaviors. The bedding was changed weekly to maintain a low-stress environment. Mice were housed in an air-conditioned room maintained at 22 ± 2 °C and 55 ± 5% relative humidity with an automated 12 h light/dark cycle (light phase: 08:00–20:00). Following a 7-day acclimatization period with ad libitum access to a standard MF diet and water, the experiment was conducted in two phases.

1. Pre-treatment phase (7 days): Mice were randomized into two groups. The IAA pre-treatment cohort (*n* = 8) received 5 mg/mL IAA dissolved in drinking water. The vehicle cohort (*n* = 16) received regular water intake. The mean body weights were matched between cohorts to ensure baseline equivalence.
2. Disease induction phase (6 days): The vehicle cohort was further divided into two groups (*n* = 8 each): a healthy control group (receiving regular water) and a DSS group (receiving 2% *w*/*v* DSS in water). The IAA pre-treatment cohort was assigned to the DSS + IAA group (*n* = 8), which received a solution containing both 2% DSS and 5 mg/mL IAA. The mean body weights were matched across all three groups.

Throughout the 6-day induction period, all mice were fed the MF diet and provided with their respective drinking solutions, ad libitum. The mice were monitored daily for clinical symptoms. The severity of DSS-induced colitis was assessed using a previously described Disease Activity Index (DAI) scoring system [68], which includes stool consistency, rectal bleeding, and weight loss. Humane endpoints were defined as the presence of severe bloody stools, marked reduction in spontaneous movement, or body weight loss exceeding 20%. To minimize stress, the food and water were replenished every 1–2 days. The average daily IAA intake (mg/kg/day) was estimated from the recorded mean water consumption (mL/day) over the 6-day period (presented in Figure 2C), the IAA concentration (5 mg/mL), and the average body weight (kg) for each group. To reduce potential confounding variables, cage placement, and all measurements and dissections were randomized. Furthermore, to minimize experimenter bias, all clinical assessments (including DAI scoring), sample collections, and subsequent biochemical analyses were performed by operators blinded to the experimental group allocations. At the end of the experiment, the mice were anesthetized with 3% isoflurane for induction and maintained under 2% isoflurane via a nose cone. Blood was collected from the abdominal vena cava into syringes containing heparin (final concentration, 50 U/mL) and aprotinin (final concentration, 500 kIU/mL). Plasma was subsequently prepared. Mice were euthanized by exsanguination via transection of the abdominal aorta and the inferior vena cava. The liver, epididymal adipose tissue, and kidneys were promptly harvested and weighed for further analyses.

### 4.3. Measurement of Plasma Parameters

Plasma was prepared by centrifuging blood samples at 2000× *g* for 10 min at 4 °C. As the primary objective of this study was to elucidate the systemic complications associated with colitis (e.g., cachexia, inflammatory anemia, and incipient renal dysfunction), plasma was selected as the most appropriate sample for these comprehensive biochemical assessments. The concentrations of various plasma parameters were subsequently measured by Oriental Yeast Co., Ltd. (Tokyo, Japan).

### 4.4. Statistical Analysis

All data are presented as the mean ± standard error (SE), and statistical significance was defined as *p* < 0.05. For comparisons of physiological and biochemical parameters among the three experimental groups (Control, DSS, and DSS + IAA), as well as for univariate comparisons of principal component scores (PC1 and PC2), a one-way analysis of variance (ANOVA) was performed, followed by Dunnett’s test for multiple comparisons. To specifically evaluate the intervention effect of IAA, pairwise comparisons between the DSS and DSS + IAA groups were conducted using Student’s *t*-test. Statistical analyses were performed using Microsoft Excel 2011 (Microsoft Corp., Redmond, WA, USA) and Statcel 4 (OMS Publishing Co., Saitama, Japan). Core multivariate and correlation structure analyses were conducted in a Python (version 3.12) environment to ensure reproducibility, utilizing the following libraries: scikit-learn (v1.3), statsmodels (v0.14), and scipy (v1.11.3). The specific analytical methods were as follows.

- Principal Component Analysis (PCA) and Multivariate Score Tests: All parameters were standardized using z-score normalization prior to PCA. Principal component scores (PC1 and PC2) were calculated to capture the major biological variations. Inter-group differences in the multivariate space defined by PC1 and PC2 were assessed using multivariate analysis of variance (MANOVA), with Wilks’ Lambda and Pillai’s trace as test statistics. Pairwise group comparisons were further evaluated using Hotelling’s T^2^ test.
- Correlation Structure Analysis: Spearman’s rank correlation coefficients were calculated between the variables within each group. Fisher’s Z-transformation was applied to assess significant differences in correlation strength between groups.

## 5. Conclusions

The present study comprehensively demonstrated that oral administration of the gut microbial metabolite IAA not only significantly alleviates intestinal inflammation in a DSS-induced colitis model but also exerts pleiotropic protective effects against severe systemic complications, including cachexia (loss of body weight and fat), inflammatory anemia, and incipient renal dysfunction. A particularly novel finding is the “food intake-independent” mechanism, whereby these therapeutic effects were achieved without a full recovery in total food intake, suggesting that IAA exerts direct metabolic regulatory actions beyond simple nutritional support. Furthermore, the observation that IAA, previously known as a uremic toxin, ameliorated early-stage renal dysfunction reveals an academically important dichotomy in its physiological effects, which appear to be stage-dependent. Multidimensional analysis using PCA statistically corroborated that IAA ameliorates the complex pathology of IBD, encompassing disease activity, systemic wasting, and renal impairment. Importantly, this amelioration does not represent a complete return to the original healthy state but rather the establishment of a novel and therapeutically beneficial physiological state. These findings present the possibility of a groundbreaking therapeutic strategy that targets gut microbial metabolites to address the systemic complications of IBD, particularly cachexia, which remains a major unmet medical need. In the future, the correlations among multiple physiological indicators and the PCA-based systemic health index developed in this study may contribute to the realization of AI-driven personalized medicine that can contribute to the identification of early warning indicators for disease progression and thresholds for determining the optimal timing of intervention.

## Figures and Tables

**Table 1:** Contribution rates (explained variance) of the first two principal components in the PCA.

Principal Component	Explained Variance Ratio (%)
PC1	49.4
PC2	15.7
PC1 + PC2	65.1

**Table 2:** Variable loadings for principal component 1 (PC1), interpreted as the “disease severity axis”.

Variable	PC1 Loading
Colon length	0.29
DAI	−0.29
Body weight	0.29
ΔBody weight	0.29
Water intake (Day 6)	0.29
Plasma Fe concentration	0.29
Food intake (Day 6)	0.29
Fat weight	0.28
Fat/Body weight	0.26
Kidney weight	0.24
Food intake (DSS period)	0.24
Water intake (DSS period)	0.14
Plasma Na concentration	−0.12
Kidney/Body weight	−0.12
Plasma Mg concentration	−0.11
Creatinine	−0.11
BUN	−0.11
Plasma K concentration	−0.10
Plasma Cl concentration	−0.09
Plasma IP concentration	−0.02
Plasma Ca concentration	−0.02 × 10^−1^

**Table 3:** Variable loadings for principal component 2 (PC2), interpreted as the “metabolic and renal function axis”.

Variable	PC2 Loading
BUN	0.45
Plasma Mg concentration	0.42
Plasma IP concentration	0.42
Plasma Ca concentration	−0.38
Kidney/Body weight	−0.33
Food intake (DSS period)	0.26
Creatinine	0.14
Kidney weight	−0.14
Water intake (DSS period)	0.13
Plasma Cl concentration	0.12
Fat/Body weight	−0.07
Plasma Na concentration	−0.07
Body weight	0.06
DAI	−0.05
Plasma K concentration	−0.04
Plasma Fe concentration	0.04
Food intake (Day 6)	0.04
Fat weight	−0.02
ΔBody weight	−0.01
Water intake (Day 6)	0.01
Colon length	−0.01 × 10^−2^

**Table 4:** Results of multivariate analysis of variance (MANOVA) using PC1 and PC2 scores.

Indicator	Value	*p* Value
Wilks’ lambda	0.13	1.89 × 10^−8^
Pillai’s trace	0.9390	1.79 × 10^−5^

**Table 5:** Pairwise group comparisons in PCA space using Hotelling’s T^2^ test.

Comparison Group	Hotelling T^2^	*F* Value	*p* Value
Cont vs. DSS	106.519	49.455	0.01 × 10^−4^
Cont vs. IAA	83.922	38.964	0.03 × 10^−4^
DSS vs. IAA	8.783	4.078	0.04

**Table 6:** One-way ANOVA results for PC1 and PC2 scores.

Component	*F* Value	*p* Value
PC1	59.74715787	2.15 × 10^−9^
PC2	1.019661616	0.37

**Table 7:** Significant changes were observed in the correlation coefficients between physiological parameters across the experimental groups.

Group 1	Group 2	Variable 1	Variable 2	Spearman_r (Variable 1)	Spearman_r (Variable 2)	Z_Statistic	*p* Value
Cont	DSS	Body weight	Food intake (DSS period)	0.83	−0.25	2.30	0.02
Cont	DSS	Body weight	K	0.40	−0.73	2.15	0.03
Cont	DSS	Body weight	Ca	0.87	−0.03	2.19	0.02
Cont	DSS	Body weight	IP	0.97	−0.16	3.57	0.03 × 10^−2^
Cont	DSS	ΔBody weight	Water intake (Day 6)	0.61	0.97	−2.34	0.01
Cont	DSS	ΔBody weight	Fat weight	0.21	0.92	−2.26	0.02
Cont	DSS	ΔBody weight	Fat/Body weight	0.14	0.90	−2.14	0.03
Cont	DSS	Food intake (DSS period)	Food intake (Day 6)	0.92	−0.03	2.66	0.07 × 10^−1^
Cont	DSS	Food intake (DSS period)	Water intake (Day 6)	0.92	−0.25	3.01	0.02 × 10^−1^
Cont	DSS	Food intake (DSS period)	Kidney weight	0.90	−0.27	2.81	0.04 × 10^−1^
Cont	DSS	Food intake (DSS period)	Kidney/ Body weight	0.85	−0.35	2.62	0.08 × 10^−1^
Cont	DSS	Food intake (DSS period)	Plasma Cl concentration	−0.49	0.74	−2.35	0.01
Cont	DSS	Food intake (Day 6)	Kidney/Body weight	0.80	−0.59	2.86	0.04 × 10^−1^
Cont	DSS	Water intake (DSS period)	Water intake (Day 6)	0.97	0.09	3.34	0.08 × 10^−2^
Cont	DSS	Water intake (DSS period	Kidney weight	0.88	0	2.18	0.02
Cont	DSS	Water intake (DSS period)	Kidney/Body weight	0.64	−0.59	2.29	0.02
Cont	DSS	Water intake (Day 6)	Colon length	0.59	0.97	−2.40	0.01
Cont	DSS	Water intake (Day 6)	Fat weight	0	0.90	−2.36	0.01
Cont	DSS	Water intake (Day 6)	Fat/Body weight	−0.23	0.88	−2.56	0.01
Cont	DSS	Fat/Body weigh	Plasma Fe concentration	−0.47	0.76	−2.40	0.01
Cont	DSS	Kidney weight	Plasma K concentration	0.56	−0.58	2.07	0.03
Cont	DSS	Kidney weight	Plasma IP concentration	0.80	−0.16	2.01	0.04
Cont	DSS	Cl	Plasma IP concentration	−0.77	0.32	−2.18	0.02
Cont	DSS	Ca	Plasma IP concentration	0.79	−0.27	2.16	0.03
Cont	IAA	Body weight	Water intake (DSS period)	0.92	0.35	2.01	0.04
Cont	IAA	Body weight	Water intake (Day 6)	0.90	−0.13	2.57	0.09 × 10^−1^
Cont	IAA	Body weight	Plasma Ca concentration	0.87	0.02	2.09	0.03
Cont	IAA	Body weight	Plasma IP concentration	0.97	0.50	2.43	0.01
Cont	IAA	ΔBody weight	Plasma Ca concentration	0.79	−0.63	2.89	0.03 × 10^−1^
Cont	IAA	Food intake (DSS period)	Water intake (DSS period)	0.90	0.04	2.29	0.02
Cont	IAA	Food intake (DSS period)	Kidney weight	0.90	−0.17	2.65	0.07 × 10^−1^
Cont	IAA	Food intake (DSS period)	Kidney/Body weight	0.85	−0.54	3.00	0.02 × 10^−1^
Cont	IAA	Food intake (DSS period)	Plasma Ca concentration	0.61	−0.57	2.16	0.03
Cont	IAA	Food intake (Day 6)	Water intake (DSS period)	0.73	−0.33	2.04	0.04
Cont	IAA	Food intake (Day 6)	BUN	−0.64	0.45	−1.97	0.04
Cont	IAA	Food intake (Day 6)	Plasma Cl concentration	−0.23	0.78	−2.05	0.03
Cont	IAA	Water intake (DSS period)	Water intake (Day 6)	0.97	0.03	3.43	0.05 × 10^−2^
Cont	IAA	Water intake (Day 6)	Kidney weight	0.90	−0.06	2.46	0.01
Cont	IAA	Water intake (Day 6)	Plasma Ca concentration	0.75	−0.41	2.25	0.02
Cont	IAA	Colon length	Plasma Ca Concentration	0.73	−0.60	2.57	0.09 × 10^−1^
Cont	IAA	Kidney/Body weight	BUN	−0.88	0.09	−2.33	0.01
Cont	IAA	BUN	Plasma K concentration	−0.44	0.71	−2.15	0.03
Cont	IAA	Creatinine	Plasma K concentration	0.57	−0.63	2.20	0.02
Cont	IAA	Plasma Fe concentration	Plasma Ca concentration	0.46	−0.88	3.00	0.02 × 10^−1^
Cont	IAA	Plasma Na concentration	Plasma Cl concentration	0.91	0.21	2.07	0.03
Cont	IAA	Plasma Ca concentration	Plasma IP concentration	0.79	−0.33	2.27	0.02
DSS	IAA	DAI	Food intake (DSS period)	0.21	−0.77	1.99	0.04
DSS	IAA	ΔBody weight	Water intake (Day 6)	0.97	0.46	2.69	0.07 × 10^−1^
DSS	IAA	Food intake (DSS period)	Creatinine	0.66	−0.71	2.69	0.07 × 10^−1^
DSS	IAA	Food intake (DSS period)	Plasma Fe concentration	−0.49	0.85	−2.87	0.04 × 10^−1^
DSS	IAA	Water intake (Day 6)	Colon length	0.97	−0.06	3.59	0.03 × 10^−2^
DSS	IAA	Water intake (Day 6)	Fat weight	0.90	0.17	2.08	0.03
DSS	IAA	Water intake (Day 6)	Fat/Body weight	0.88	0.09	2.03	0.04
DSS	IAA	Kidney weight	Plasma Fe concentration	0.71	−0.34	1.98	0.04
DSS	IAA	Kidney weight	Plasma K concentration	−0.58	0.59	−2.14	0.03
DSS	IAA	Kidney/Body weight	Plasma Cl concentration	−0.59	0.52	−1.99	0.04
DSS	IAA	BUN	Creatinine	0.49	−0.76	2.46	0.01
DSS	IAA	BUN	Plasma IP concentration	0.66	0.56	2.27	0.02
DSS	IAA	Creatinine	Plasma IP concentration	0.87	0.08	2.02	0.04
DSS	IAA	Creatinine	Plasma Mg concentration	0.83	−0.12	2.13	0.03
DSS	IAA	Plasma Fe concentration	Plasma Ca concentration	−0.05	−0.88	2.10	0.03

## Data Availability

The original contributions presented in this study are included in this article. Further inquiries can be directed to the corresponding author.

## References

[1] Nakase H., Uchino M., Shinzaki S., Matsuura M., Matsuoka K., Kobayashi T., Saruta M., Hirai F., Hata K., Hiraoka S. (2021). Evidence-based clinical practice guidelines for inflammatory bowel disease 2020. J. Gastroenterol..

[2] Kirsner J.B. (1979). The local and systemic complications of inflammatory bowel disease. JAMA.

[3] Rogler G., Singh A., Kavanaugh A., Rubin D.T. (2021). Extraintestinal Manifestations of Inflammatory Bowel Disease: Current Concepts, Treatment, and Implications for Disease Management. Gastroenterology.

[4] Faggiani I., Fanizza J., D’Amico F., Allocca M., Zilli A., Parigi T.L., Barchi A., Danese S., Furfaro F. (2024). Extraintestinal Manifestations in Inflammatory Bowel Disease: From Pathophysiology to Treatment. Biomedicines.

[5] Liu L.F., Fan Y.W., Lv Y., Liu Z.X., Dai X.C. (2025). Strategies for assessing and preventing cardiovascular disease risk in inflammatory bowel disease patients: A meta-analysis and meta-regression and bibliometric review. PLoS ONE.

[6] Thomas D.R., Huangfu G., Yeaman F., Sukudom S., Lan N.S.R., Dwivedi G., Thin L. (2025). Association between inflammatory bowel disease, current therapies, and cardiovascular events: A review and meta-analysis of data from 2.2 million individuals. J. Crohn’s Colitis.

[7] Jaiswal V., Batra N., Dagar M., Butey S., Huang H., Chia J.E., Naz S., Endurance E.O., Raj N., Patel S. (2023). Inflammatory bowel disease and associated cardiovascular disease outcomes: A systematic review. Medicine.

[8] Ji Y., Gao Y., Chen H., Yin Y., Zhang W. (2019). Indole-3-Acetic Acid Alleviates Non-alcoholic Fatty Liver Disease in Mice via Attenuation of Hepatic Lipogenesis, and Oxidative and Inflammatory Stress. Nutrients.

[9] Ding Y., Yanagi K., Yang F., Callaway E., Cheng C., Hensel M.E., Menon R., Alaniz R.C., Lee K., Jayaraman A. (2024). Oral supplementation of gut microbial metabolite indole-3-acetate alleviates diet-induced steatosis and inflammation in mice. eLife.

[10] Zhong L., Boopathi S., Purushothaman B., Tu Q., Zhang Y. (2025). Gut microbiota-indole-3-acetic acid axis in cancer: Dual functions, mechanistic insights, and therapeutic potential. Microbiol. Res..

[11] Tintelnot J., Xu Y., Lesker T.R., Schonlein M., Konczalla L., Giannou A.D., Pelczar P., Kylies D., Puelles V.G., Bielecka A.A. (2023). Microbiota-derived 3-IAA influences chemotherapy efficacy in pancreatic cancer. Nature.

[12] Yang J., Wang H., Yan J., Sun J., Wang Y., Huang G., Zhang F., Cao H., Li D. (2025). Biotherapeutic potential of gut microbiota-derived indole-3-acetic acid. Crit. Rev. Microbiol..

[13] Ng S.C., Shi H.Y., Hamidi N., Underwood F.E., Tang W., Benchimol E.I., Panaccione R., Ghosh S., Wu J.C.Y., Chan F.K.L. (2017). Worldwide incidence and prevalence of inflammatory bowel disease in the 21st century: A systematic review of population-based studies. Lancet.

[14] Wang R., Li Z., Liu S., Zhang D. (2023). Global, regional and national burden of inflammatory bowel disease in 204 countries and territories from 1990 to 2019: A systematic analysis based on the Global Burden of Disease Study 2019. BMJ Open.

[15] Zhang Y., Chung H., Fang Q.W., Xu Y.R., Zhang Y.J., Nakajo K., Wong I.C.K., Leung W.K., Qiu H., Li X. (2025). Current and forecasted 10-year prevalence and incidence of inflammatory bowel disease in Hong Kong, Japan, and the United States. World J. Gastroenterol..

[16] Ungaro R., Mehandru S., Allen P.B., Peyrin-Biroulet L., Colombel J.F. (2017). Ulcerative colitis. Lancet.

[17] Hazel K., O’Connor A. (2020). Emerging treatments for inflammatory bowel disease. Ther. Adv. Chronic Dis..

[18] Caballero Mateos A.M., Cañadas de la Fuente G.A., Gros B. (2025). Paradigm Shift in Inflammatory Bowel Disease Management: Precision Medicine, Artificial Intelligence, and Emerging Therapies. J. Clin. Med..

[19] Caron B., Habert A., Bonsack O., Camara H., Jeanbert E., Parigi T.L., Netter P., Danese S., Peyrin-Biroulet L. (2024). Difficult-to-treat inflammatory bowel disease: Effectiveness and safety of 4th and 5th lines of treatment. United Eur. Gastroenterol. J..

[20] Moss A.C. (2022). Approach to Treatment Failure in Inflammatory Bowel Disease. Gastroenterol. Hepatol..

[21] State M., Negreanu L. (2023). Defining the Failure of Medical Therapy for Inflammatory Bowel Disease in the Era of Advanced Therapies: A Systematic Review. Biomedicines.

[22] Lee M., Chang E.B. (2021). Inflammatory Bowel Diseases (IBD) and the Microbiome-Searching the Crime Scene for Clues. Gastroenterology.

[23] Tie Y., Huang Y., Chen R., Li L., Chen M., Zhang S. (2023). Current insights on the roles of gut microbiota in inflammatory bowel disease-associated extra-intestinal manifestations: Pathophysiology and therapeutic targets. Gut Microbes.

[24] Shaheen N., Miao J., Xia B., Zhao Y., Zhao J. (2025). Multifaceted Role of Microbiota-Derived Indole-3-Acetic Acid in Human Diseases and Its Potential Clinical Application. FASEB J..

[25] Lopetuso L.R., Deleu S., Puca P., Abreu M.T., Armuzzi A., Barbara G., Caprioli F., Chieng S., Costello S.P., Damiani A. (2025). Guidance for Fecal Microbiota Transplantation Trials in Ulcerative Colitis: The Second ROME Consensus Conference. Inflamm. Bowel Dis..

[26] Lamas B., Richard M.L., Leducq V., Pham H.P., Michel M.L., Da Costa G., Bridonneau C., Jegou S., Hoffmann T.W., Natividad J.M. (2016). CARD9 impacts colitis by altering gut microbiota metabolism of tryptophan into aryl hydrocarbon receptor ligands. Nat. Med..

[27] Li M., Ding Y., Wei J., Dong Y., Wang J., Dai X., Yan J., Chu F., Zhang K., Meng F. (2024). Gut microbiota metabolite indole-3-acetic acid maintains intestinal epithelial homeostasis through mucin sulfation. Gut Microbes.

[28] Monteleone I., Rizzo A., Sarra M., Sica G., Sileri P., Biancone L., MacDonald T.T., Pallone F., Monteleone G. (2011). Aryl hydrocarbon receptor-induced signals up-regulate IL-22 production and inhibit inflammation in the gastrointestinal tract. Gastroenterology.

[29] Moutusy S.I., Ohsako S. (2024). Gut Microbiome-Related Anti-Inflammatory Effects of Aryl Hydrocarbon Receptor Activation on Inflammatory Bowel Disease. Int. J. Mol. Sci..

[30] Shiomi Y., Nishiumi S., Ooi M., Hatano N., Shinohara M., Yoshie T., Kondo Y., Furumatsu K., Shiomi H., Kutsumi H. (2011). GCMS-based metabolomic study in mice with colitis induced by dextran sulfate sodium. Inflamm. Bowel Dis..

[31] Ji T., Xu C., Sun L., Yu M., Peng K., Qiu Y., Xiao W., Yang H. (2015). Aryl Hydrocarbon Receptor Activation Down-Regulates IL-7 and Reduces Inflammation in a Mouse Model of DSS-Induced Colitis. Dig. Dis. Sci..

[32] Vyhlídalová B., Krasulová K., Pečinková P., Marcalíková A., Vrzal R., Zemánková L., Vančo J., Trávníček Z., Vondráček J., Karasová M. (2020). Gut Microbial Catabolites of Tryptophan Are Ligands and Agonists of the Aryl Hydrocarbon Receptor: A Detailed Characterization. Int. J. Mol. Sci..

[33] Chowdhury M.M.I., Kurata K., Yuasa K., Koto Y., Nishimura K., Shimizu H. (2021). Suppression of TNFα expression induced by indole-3-acetic acid is not mediated by AhR activation in Caco-2 cells. Biosci. Biotechnol. Biochem..

[34] Chowdhury M.M.I., Tomii A., Ishii K., Tahara M., Hitsuda Y., Koto Y., Kurata K., Yuasa K., Nishimura K., Shimizu H. (2021). TLR4 may be a novel indole-3-acetic acid receptor that is implicated in the regulation of CYP1A1 and TNFα expression depending on the culture stage of Caco-2 cells. Biosci. Biotechnol. Biochem..

[35] Tomii A., Higa M., Naito K., Kurata K., Kobayashi J., Takei C., Yuasa K., Koto Y., Shimizu H. (2023). Activation of the TLR4-JNK but not the TLR4-ERK pathway induced by indole-3-acetic acid exerts anti-proliferative effects on Caco-2 cells. Biosci. Biotechnol. Biochem..

[36] Qu X., Song Y., Li Q., Xu Q., Li Y., Zhang H., Cheng X., Mackay C.R., Wang Q., Liu W. (2024). Indole-3-acetic acid ameliorates dextran sulfate sodium-induced colitis via the ERK signaling pathway. Arch. Pharm. Res..

[37] Ji Y., Yin W., Liang Y., Sun L., Yin Y., Zhang W. (2020). Anti-Inflammatory and Anti-Oxidative Activity of Indole-3-Acetic Acid Involves Induction of HO-1 and Neutralization of Free Radicals in RAW264.7 Cells. Int. J. Mol. Sci..

[38] Fink C., Karagiannides I., Bakirtzi K., Pothoulakis C. (2012). Adipose tissue and inflammatory bowel disease pathogenesis. Inflamm. Bowel Dis..

[39] Gonçalves P., Magro F., Martel F. (2015). Metabolic inflammation in inflammatory bowel disease: Crosstalk between adipose tissue and bowel. Inflamm. Bowel Dis..

[40] Karaskova E., Velganova-Veghova M., Geryk M., Foltenova H., Kucerova V., Karasek D. (2021). Role of Adipose Tissue in Inflammatory Bowel Disease. Int. J. Mol. Sci..

[41] Eder P., Adler M., Dobrowolska A., Kamhieh-Milz J., Witowski J. (2019). The Role of Adipose Tissue in the Pathogenesis and Therapeutic Outcomes of Inflammatory Bowel Disease. Cells.

[42] Lee C., Kim S., Kim B., Holzapfel W.H., Hyun C.K. (2023). Disturbance of lipid metabolism in germ-free mice transplanted with gut microbiota of DSS-induced colitis mice. PLoS ONE.

[43] Aslam T., Mehmood A. (2023). Prevalence and Risk Factors of Anemia in Inflammatory Bowel Diseases: A Case-Control Study. Cureus.

[44] Eriksson C., Henriksson I., Brus O., Zhulina Y., Nyhlin N., Tysk C., Montgomery S., Halfvarson J. (2018). Incidence, prevalence and clinical outcome of anaemia in inflammatory bowel disease: A population-based cohort study. Aliment. Pharmacol. Ther..

[45] Carrier J., Aghdassi E., Platt I., Cullen J., Allard J.P. (2001). Effect of oral iron supplementation on oxidative stress and colonic inflammation in rats with induced colitis. Aliment. Pharmacol. Ther..

[46] Mahalhal A., Williams J.M., Johnson S., Ellaby N., Duckworth C.A., Burkitt M.D., Liu X., Hold G.L., Campbell B.J., Pritchard D.M. (2018). Oral iron exacerbates colitis and influences the intestinal microbiome. PLoS ONE.

[47] Zhang Y., Yin L., Zeng X., Li J., Yin Y., Wang Q., Li J., Yang H. (2022). Dietary High Dose of Iron Aggravates the Intestinal Injury but Promotes Intestinal Regeneration by Regulating Intestinal Stem Cells Activity in Adult Mice With Dextran Sodium Sulfate-Induced Colitis. Front. Vet. Sci..

[48] Loveikyte R., Bourgonje A.R., van Goor H., Dijkstra G., van der Meulen-de Jong A.E. (2023). The effect of iron therapy on oxidative stress and intestinal microbiota in inflammatory bowel diseases: A review on the conundrum. Redox Biol..

[49] Liang L., Xiong Q., Kong J., Tian C., Miao L., Zhang X., Du H. (2021). Intraperitoneal supplementation of iron alleviates dextran sodium sulfate-induced colitis by enhancing intestinal barrier function. Biomed. Pharmacother..

[50] Shanmugam N.K., Ellenbogen S., Trebicka E., Wang L., Mukhopadhyay S., Lacy-Hulbert A., Gallini C.A., Garrett W.S., Cherayil B.J. (2012). Tumor necrosis factor α inhibits expression of the iron regulating hormone hepcidin in murine models of innate colitis. PLoS ONE.

[51] Kautz L., Jung G., Valore E.V., Rivella S., Nemeth E., Ganz T. (2014). Identification of erythroferrone as an erythroid regulator of iron metabolism. Nat. Genet..

[52] Bessman N.J., Mathieu J.R.R., Renassia C., Zhou L., Fung T.C., Fernandez K.C., Austin C., Moeller J.B., Zumerle S., Louis S. (2020). Dendritic cell-derived hepcidin sequesters iron from the microbiota to promote mucosal healing. Science.

[53] Han X., Xu Z., Chang Y., Li H., Hu S., Chang S., Liu Y., Yu C., Tang T., Li Y. (2024). Concurrent chronic kidney disease in patients with inflammatory bowel disease, a systematic review and meta-analysis. Front. Med..

[54] Zadora W., Innocenti T., Verstockt B., Meijers B. (2024). Chronic Kidney Disease in Inflammatory Bowel Disease: A Systematic Review and Meta-analysis. J. Crohn’s Colitis.

[55] Yang Y., Ludvigsson J.F., Forss A., Faucon A.L., Faye A.S., Olén O., Sjölander A., Carrero J.J. (2024). Risk of Kidney Failure in Patients With Inflammatory Bowel Disease Undergoing Colectomy: A Nationwide Cohort Study. Clin. Gastroenterol. Hepatol..

[56] Zhang H., Huang Y., Zhang J., Su H., Ge C. (2023). Causal effects of inflammatory bowel diseases on the risk of kidney stone disease: A two-sample bidirectional mendelian randomization. BMC Urol..

[57] Caillard P., Bennis Y., Six I., Bodeau S., Kamel S., Choukroun G., Maizel J., Titeca-Beauport D. (2022). The Role of Gut-Derived, Protein-Bound Uremic Toxins in the Cardiovascular Complications of Acute Kidney Injury. Toxins.

[58] Daneshamouz S., Eduok U., Abdelrasoul A., Shoker A. (2021). Protein-bound uremic toxins (PBUTs) in chronic kidney disease (CKD) patients: Production pathway, challenges and recent advances in renal PBUTs clearance. NanoImpact.

[59] Papi S., Ahmadvand H., Sotoodehnejadnematalahi F., Yaghmaei P. (2022). The Protective Effects of Indole-Acetic Acid on the Renal Ischemia-Reperfusion Injury via Antioxidant and Antiapoptotic Properties in A Rat Model. Iran. J. Kidney Dis..

[60] Alhusaini A.M., Sarawi W., Mukhtar N., Aljubeiri D., Aljarboa A.S., Alduhailan H., Almutairi F., Mohammad R., Atteya M., Hasan I. (2024). Role of Nrf2/HO-1 and cytoglobin signaling in the protective effect of indole-3-acetic acid and chenodeoxycholic acid against kidney injury induced by valproate. Heliyon.

[61] Jing J., Yan X., Wang L., Zhang Y., Qi W., Xi J., Hao Z. (2025). Gut microbiota-derived indole-3-acetic acid ameliorates calcium oxalate renal stone formation via AHR/NF-κB axis. Urolithiasis.

[62] Li M., Han X., Sun L., Liu X., Zhang W., Hao J. (2024). Indole-3-acetic acid alleviates DSS-induced colitis by promoting the production of R-equol from Bifidobacterium pseudolongum. Gut Microbes.

[63] Chassaing B., Aitken J.D., Malleshappa M., Vijay-Kumar M. (2014). Dextran sulfate sodium (DSS)-induced colitis in mice. Curr. Protoc. Immunol..

[64] Oh S.Y., Cho K.A., Kang J.L., Kim K.H., Woo S.Y. (2014). Comparison of experimental mouse models of inflammatory bowel disease. Int. J. Mol. Med..

[65] Yang C., Merlin D. (2024). Unveiling Colitis: A Journey through the Dextran Sodium Sulfate-induced Model. Inflamm. Bowel Dis..

[66] Wang J., Hao Y., Yang Y., Zhang Y., Xu C., Yang R. (2025). Gut microbiota derived indole-3-acetic acid ameliorates precancerous inflammatory intestinal milieu to inhibit tumorigenesis through IL-35. J. Immunother. Cancer.

[67] Gong Q., Qu X., Zhao Y., Zhang X., Cao S., Wang X., Song Y., Mackay C.R., Wang Q. (2024). Indole-3-Acetic Acid Esterified with Waxy, Normal, and High-Amylose Maize Starches: Comparative Study on Colon-Targeted Delivery and Intestinal Health Impact. Nutrients.

[68] Murthy S.N., Cooper H.S., Shim H., Shah R.S., Ibrahim S.A., Sedergran D.J. (1993). Treatment of dextran sulfate sodium-induced murine colitis by intracolonic cyclosporin. Dig. Dis. Sci..

